# Dual Urea Cycle Dysfunction Caused by Coexisting Argininosuccinic Aciduria and Citrin Deficiency: Clinical, Biochemical, and Molecular Insights From an Exceptional Case

**DOI:** 10.1002/jmd2.70129

**Published:** 2026-10-04

**Authors:** Mouna Zribi, Mariem Bhouri, Safa Khatrouch, Hela Boudabous, Johannes Häberle, Mohamed Slim Abdelmoula, Amel Ben Chehida

**Affiliations:** ^1^ Department of Pediatrics and Inherited Metabolic Diseases La Rabta Hospital Tunis Tunisia; ^2^ Faculty of Medicine of Tunis University Tunis El Manar Tunis Tunisia; ^3^ Research Laboratory LR12SP02: “Inherited Metabolic Diseases, Investigation and Management” Tunis Tunisia; ^4^ Division of Metabolism & Children's Research University Children's Hospital Zurich Switzerland

**Keywords:** argininosuccinic aciduria, citrin deficiency, multilocus pathogenic variation, next‐generation sequencing, urea cycle disorder

## Abstract

Dual inherited defects affecting urea cycle function are exceptionally rare. We report the first case, to our knowledge, of the coexistence of argininosuccinic aciduria (ASA) and citrin deficiency (CD). A girl born to consanguineous Tunisian parents presented at 6 months of age with recurrent seizures, vomiting, developmental delay, and severe hyperammonemic encephalopathy. Laboratory investigations revealed hyperammonemia, respiratory alkalosis, transaminitis, dyslipidemia, hyperglutaminemia, and mild hypercitrullinemia. Brain imaging showed delayed myelination and basal ganglia abnormalities. Management involved arginine supplementation, ammonia scavengers, protein restriction and antiepileptic drugs. The patient died at 33 months of age from severe hyperammonemia and lactic acidosis. Next‐generation sequencing identified two homozygous variants affecting distinct urea cycle–related genes: *The known pathogenic ASL* variant c.1153C>T p.(Arg385Cys), responsible for ASA, and a previously unreported likely pathogenic *SLC25A13* variant, c.1474C>T, p.(Arg492Trp), consistent with CD. Segregation analysis confirmed biallelic inheritance of both variants. This unique association illustrates how multilocus pathogenic variation can generate an atypical phenotype. From a pathophysiological perspective, CD may aggravate urea cycle dysfunction by reducing cytosolic aspartate availability and ASS1 activity, while ASL deficiency blocks the downstream conversion of argininosuccinate, resulting in a profound impairment of ureagenesis. This dual defect creates diagnostic and therapeutic challenges, highlighting the importance of integrating comprehensive biochemical investigations with molecular testing to enable accurate diagnosis and personalized metabolic management.

## Key Points

- The coexistence of argininosuccinic aciduria and citrin deficiency can profoundly modify the clinical and biochemical expression of urea cycle dysfunction.
- This case highlights the importance of integrating detailed metabolic investigations with next‐generation sequencing to diagnose atypical hyperammonemic disorders and optimize personalized management.

## 1 Introduction

The urea cycle (UC) is an essential metabolic pathway for the detoxification of ammonia. It involves six enzymes and two transporters (including citrin) that all act in coordination to convert ammonia into urea. Genetic deficiencies affecting any enzyme or transporter involved in the UC can result in a urea cycle defect (UCD), characterized by hyperammonemia, a potentially life‐threatening condition, particularly in a developing brain [1].

The true incidence of UCDs has been estimated to range from 1 in 35 000 to 1 in 52 000 live births [2]. It is likely underestimated, as neonatal‐onset forms are often associated with early mortality and diagnostic investigations are not systematically performed in affected infants [3]. Furthermore, in countries like Tunisia, the lack of a systematic national expanded newborn screening program for inborn errors of metabolism also contributes to the underdiagnosis and delayed clinical recognition of these conditions.

To the best of our knowledge, only two cases of dual deficiency in UC enzymes have been reported since 1979 [4].

Here, we report the phenotypic and genotypic characteristics of a Tunisian child affected by an exceptionally rare dual UCD caused by the coexistence of argininosuccinate lyase (ASL) (EC 4.3.2.2) deficiency and citrin deficiency (CD), highlighting the diagnostic challenges, the complex pathophysiology and therapeutic dilemmas of this unique comorbidity.

## 2 Case Presentation and Results

The patient was a 31‐month‐old Tunisian female born to first‐cousin parents. One of her paternal cousins had been followed since the age of 8 months for failure to thrive of an unknown etiology. Pregnancy and delivery were uneventful, except for a moderate asymmetric intrauterine growth restriction (birth weight = 2300 g). The child was admitted to the neonatal unit for ABO incompatibility–related jaundice, requiring phototherapy and blood transfusion. No newborn screening was performed. Since the age of 6 months, she had recurrent hospitalizations for polymorphic seizures, associated with a mild psychomotor delay that became obvious at the age of 18 months. She was managed with sodium valproate and clonazepam. Moreover, she had a poor appetite and recurrent vomiting, without evidence of particular food preference or avoidance. She presented at the age of 31 months with status epilepticus. Physical examination noted severe acute malnutrition, with a weight below 70% of median expected weight‐for‐height based on standard WHO growth charts, with a failure to thrive (−3 SD), somnolence, hyporeactivity, global hypotonia, tachypnea and hepatomegaly. Biochemical investigations showed normal blood sugar, calcium and magnesium levels, severe hyperammonemia (443 μmol/L, normal value (NV) < 70 μmol/L), respiratory alkalosis (pH = 7.48, pCO_2_ = 29 mmHg, HCO_3_^−^ = 21.6 mmol/L), elevated liver transaminases (4× ULN), hypercholesterolemia (6.05 mmol/L; NV < 4.4 mmol/L) and hypertriglyceridemia (2.65 mmol/L; NV < 0.85 mmol/L), with milky serum.

She was managed with intravenous clonazepam, high intravenous 10% glucose infusion (6 mL/kg/h, equivalent to 10 mg/kg/min), ammonia scavengers (sodium benzoate 400 mg/kg/d, carglumic acid 250 mg/kg/d), and arginine (250 mg/kg/d). Brain CT was unremarkable, while MRI showed delayed white matter myelination and basal ganglia involvement. EEG revealed generalized paroxysmal abnormalities on a poorly organized background rhythm. Urine organic acid analysis showed no orotic acid accumulation. Amino acid profile from dried blood spots demonstrated mildly increased levels of citrulline, glutamine, and aspartic acid, with normal levels of arginine (Table 1).

**TABLE 1:** Amino acid profiles.

	Citrullin	Glutamine	Aspartic acid	Ornithine	Alanine	Tyrosine	Valine	Arginine	Phenylalanine	Methionine
Result (μmol/L)	127	441	75	125	362	29	71	23	36	11
Normal range (μmol/L)	8–47	17–78	1–17	20–160	120–600	35–107	132–480	4–30	26–98	9–35
Variation	↑	↑	↑	N	N	↓	↓	N	N	N

Plasma ammonia normalized within 5 days. Subsequent management included a high‐calorie (1000 kcal/day) and carbohydrate‐rich diet (60% of total energy intake; 150 g/day) combined with protein restriction of 10 g/day (equivalent to 0.95 g/kg/day based on a body weight of 10.5 kg). Under these nutritional interventions, while ammonia concentrations transiently remained in the normal range, the patient's clinical condition progressively deteriorated. Concurrently, plasma lactate concentrations worsened, rising from 1.6 mmol/L to 3.4 mmol/L and subsequently to 5.2 mmol/L, which prompted a reduction in carbohydrate and caloric intake. Ultimately, the patient passed away at 33 months of age following an episode of acute encephalopathy characterized by severe hyperammonemia (615 μmol/L) and lactic acidosis (pH = 7.04; pCO_2_ = 19 mmHg; HCO_3_^−^ = 5.10 mmol/L; lactate = 13.2 mmol/L). (Supplementary Table S1).

Next‐generation sequencing using a UCD gene panel identified two homozygous variants affecting distinct UC‐related genes: *the known pathogenic ASL* (OMIM#608310) variant, c.1153C>T p.(Arg385Cys), causing argininosuccinic aciduria (ASA) (OMIM#207900) and a previously unreported likely pathogenic *SLC25A13* (OMIM#603859) variant, c.1474C>T p.(Arg492Trp), consistent with CD (OMIM#603471, #605814). Segregation analysis confirmed biallelic inheritance, with both parents being heterozygous carriers for both variants. Antenatal diagnosis was performed in a subsequent pregnancy and identified a heterozygous fetus for the p.(Arg385Cys) variant in the *ASL* gene. The p.(Arg492Trp) variant in the *SLC25A13 gene* was not detected.

## 3 Discussion

To the best of our knowledge, the present case is the first case in the literature to report the coexistence of two functionally consecutive and interrelated steps of UCD. ASA is usually considered the second most common UCD after ornithine transcarbamylase deficiency, accounting for 16% of all UCDs. Its prevalence has been estimated to range from 1 in 70 000 to 1 in 218 000 live births, whereas CD has an estimated prevalence of 1 in 100 000 live births in Japan, where it was originally described [5, 6]. While national epidemiological registries for dual UCDs are currently lacking due to their extreme rarity, high rates of parental consanguinity in Tunisia (estimated at 30%) significantly increase the baseline likelihood of co‐inheriting distinct autosomal recessive mutations, leading to complex multilocus genomic phenotypes, as previously outlined [7].

The case described here has several specific features that result in a complex clinical and biochemical phenotype and give rise to distinct therapeutic challenges.

From a pathophysiological perspective, the unique feature of this case lies in the convergence of two genetic defects affecting UC: a primary enzymatic deficiency and a defect of a mitochondrial transporter. Indeed, CD impairs the export of aspartate from mitochondria, resulting in reduced cytosolic aspartate availability. As aspartate is an essential substrate for argininosuccinate synthesis by argininosuccinate synthetase (ASS1, EC 6.3.4.5), citrin deficiency may further compromise UC flux [8, 9]. Moreover, a marked reduction in cytosolic ASS1 expression has been observed in CD [10] (Figure 1).

**FIGURE 1:**
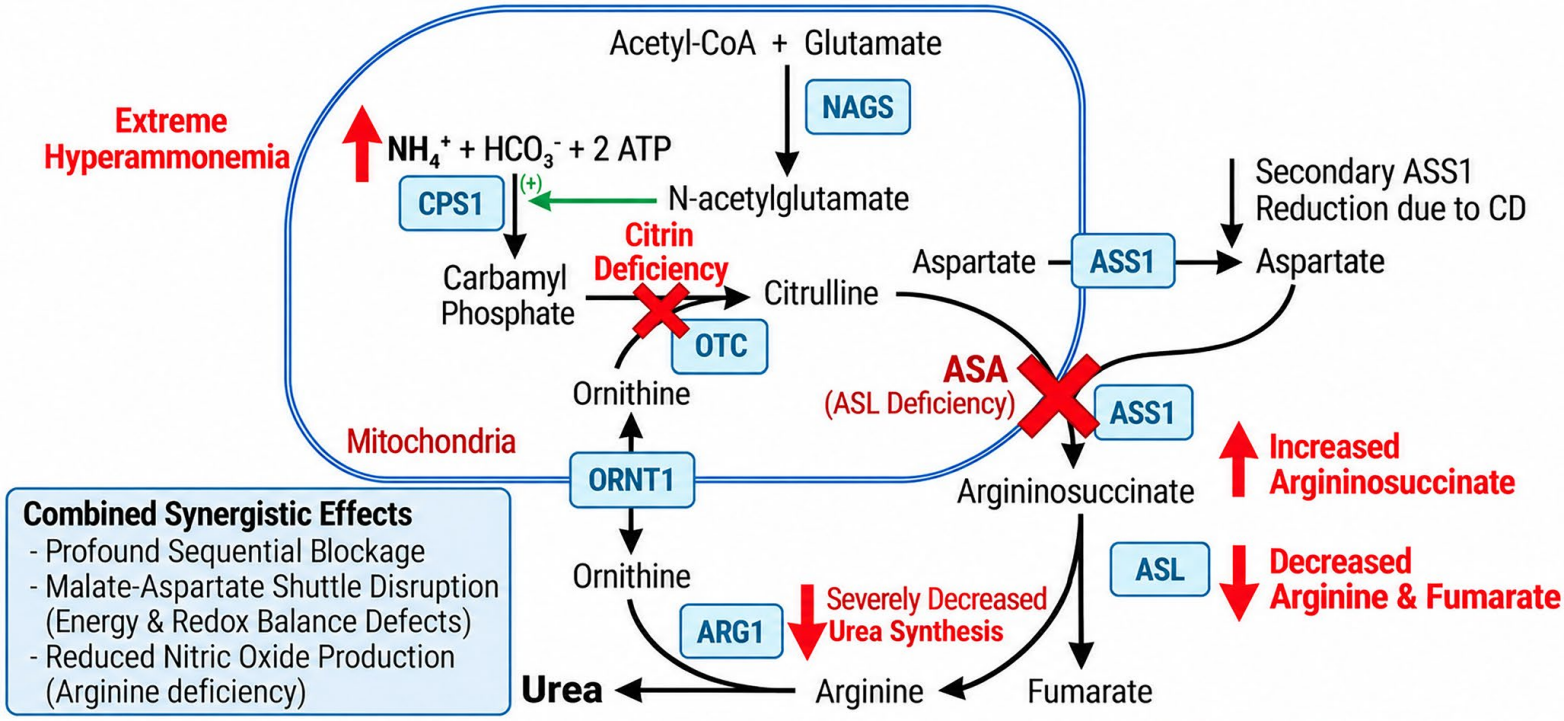
Pathophysiological consequences of dual urea cycle defect (Citrin deficiency and Argininosuccinic aciduria) on the urea cycle. Annotations in red represent the double deficiency state. Red “X” marks indicate the sites of Citrin deficiency and argininosuccinate lyase (ASL) deficiency. Up and down arrows indicate corresponding metabolite changes and cycle flow disruptions.

In contrast, ASA results from a primary deficiency of ASL, the enzyme immediately downstream of ASS1 in the UC. ASL deficiency impairs the conversion of argininosuccinate to arginine and fumarate [6]. Thus, the concurrence of these two defects is expected to have an additive effect on ureagenesis by creating a profound sequential disruption of UC (Figure 1). This dual impairment likely contributes to the severe hyperammonemia and the hyperglutaminemia observed in our patient.

At the clinical level, ASA encompasses a broad spectrum of manifestations. While epilepsy and neurodevelopmental impairment are widely reported [11, 12], our patient developed seizures at an unusually early age of 6 months, which is earlier than typically observed in pediatric cohorts [12, 13]. Notably, sodium valproate was initially prescribed to control these refractory seizures before the metabolic diagnosis was established. Although commonly used for pediatric epilepsy, valproate is strictly contraindicated in UCDs as it inhibits N‐acetylglutamate synthase (NAGS) and mitochondrial fatty acid oxidation, thereby potentially exacerbating hyperammonemic crises. Regarding additional features, she also exhibited hepatic involvement, a feature reported in nearly half of ASA cases. However, she lacked the muscular, behavioral, cardiovascular (including hypertension), renal, or dermatological manifestations often described in the literature [11].

CD is characterized by an age‐dependent clinical presentation [9]. In our patient, the phenotype appears to result from a complex overlap of both metabolic defects. While the early‐onset epilepsy, recurrent vomiting, developmental delay, transaminitis, and severe hyperammonemia are highly characteristic of ASA [11, 13], her failure to thrive and dyslipidemia strongly point toward the concomitant contribution of CD, despite the absence of a history of neonatal intrahepatic cholestasis [9]. This unique dual‐pathology combination likely accounts for the exceptionally complex and severe clinical course observed in this case.

Beyond the hyperammonemia, additional pathophysiological mechanisms may contribute to the clinical severity observed in our patient. In CD, disruption of the malate–aspartate NADH shuttle results in an altered cytosolic NADH/NAD+ redox balance in the liver, which can lead to the impairment of hepatic glycolysis, hepatocellular energy deficit and hyperlactacidemia [9], as observed in our patient. ASL deficiency results in impaired ureagenesis, systemic arginine deficiency and reduced nitric oxide production [6, 14]. These dual metabolic consequences (Figure 2) may therefore have additive or synergistic effects on organ dysfunction. Thus, the coexistence of these two disorders could account for the unusually early and severe neurological presentation and fatal outcome observed in our patient, compared with what would be expected in either disorder occurring in isolation.

**FIGURE 2:**
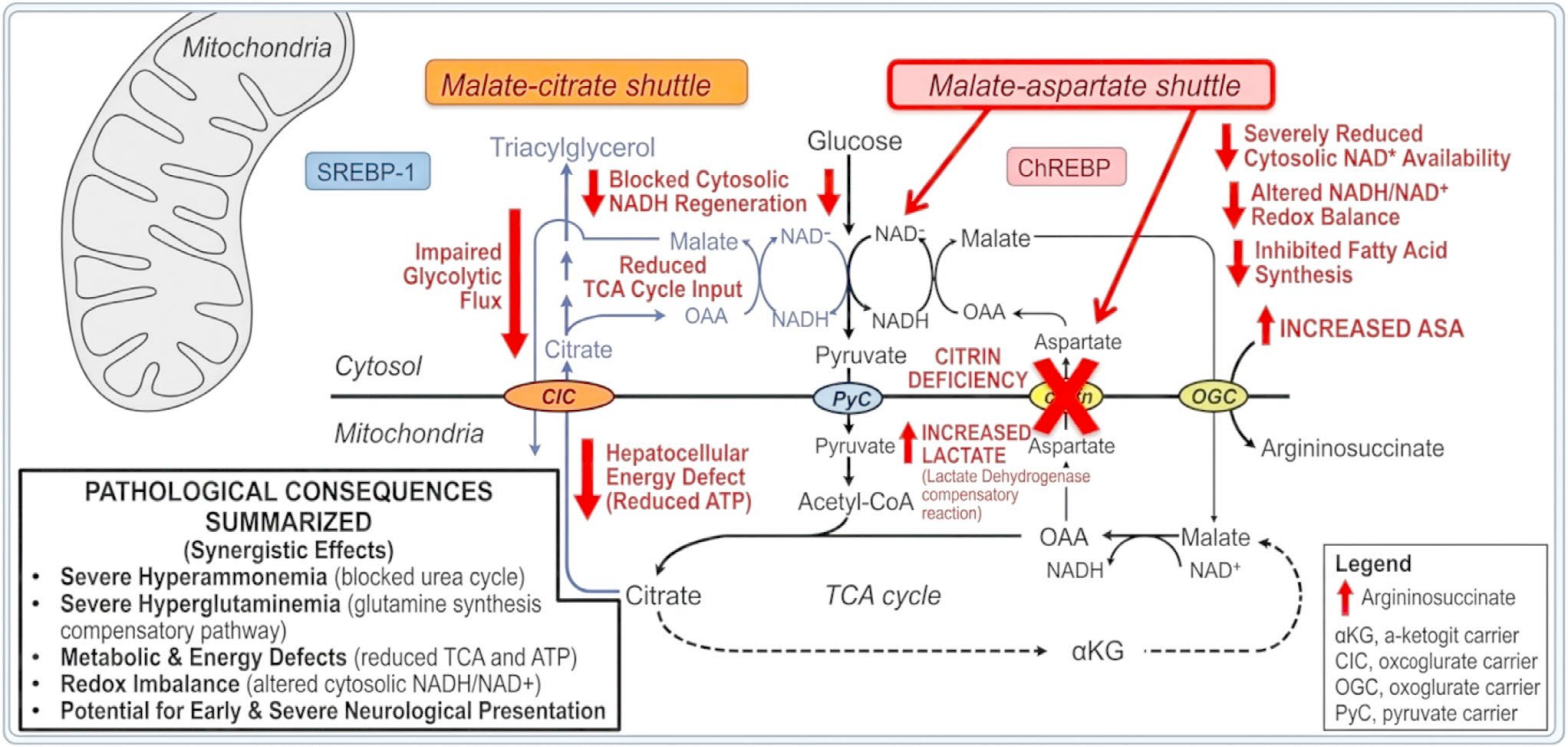
Consequences of the double deficiency on malate–aspartate NADH shuttle, energetic metabolism and cytosolic redox state. Red X mark indicates deficiency. Annotations in red represent the double deficiency state.

The biochemical profile of our patient combined features typically observed in UCD, including hyperammonemia, respiratory alkalosis, and transaminitis [1, 2], alongside dyslipidemia, a finding that may reflect the coexisting CD [9]. Mild hypercitrullinemia and hyperglutaminemia were consistent with impaired UC function, but did not clearly indicate an isolated ASL deficiency. A notable limitation of our report is the unavailability of quantitative arginosuccinate levels due to analytical constraints in our resource‐limited setting. Furthermore, plasma amino acid profiling was performed after the initiation of arginine supplementation, which likely replenished the circulating arginine pool, thereby masking the typical hypoargininemia expected in ASL deficiency. In addition, because CD limits cytosolic aspartate availability and consequently impairs argininosuccinate synthesis [9, 10], argininosuccinate accumulation was expectedly less pronounced than typically observed in an isolated ASL defect [11, 14]. The coexistence of both defects thus modified the classical metabolic signature of each individual disease. This demonstrates how early emergency metabolic interventions can alter characteristic key biomarker profiles, underscoring the critical role of next‐generation sequencing in establishing a definitive diagnosis when biochemical findings are equivocal or atypical.

Next‐generation sequencing in the present case identified missense variants in two distinct genes involved in UCDs; the patient was homozygous for both variants. The variant c.1153C>T, p.(Arg385Cys) in exon 15 of the *ASL* gene has been previously reported in ASA [15].

According to available literature, the novel *SLC25A13* variant c.1474C>T p.(Arg492Trp) identified in our patient has not been previously reported. Although functional expression studies were not performed to formally validate the novel *SLC25A13* variant, its pathogenicity is strongly supported by multiple lines of evidence: high deleterious scores across in silico prediction algorithms, high evolutionary conservation of the Arg492 residue, co‐segregation with the phenotype in the consanguineous pedigree, and an exact match with the patient's biochemical features (dyslipidemia, energy failure, severe lactic acidosis under carbohydrate load). Consequently, this variant was classified as likely pathogenic according to ACMG/AMP guidelines, further supported by its extremely low allele frequency in public population databases, including gnomAD. Segregation analysis confirmed the biallelic inheritance of both variants, as both parents, who were first cousins, were heterozygous carriers, thereby illustrating the profound impact of parental consanguinity on the co‐occurrence of these two rare autosomal recessive disorders in this family.

Regarding management, the comorbidity of two UCDs poses an extraordinary therapeutic challenge, as established treatment guidelines for CD directly contradict those for standard UCDs such as ASA [2, 3, 14]. Indeed, CD management relies on a high‐protein, high‐fat diet with strict carbohydrate restriction and medium‐chain triglycerides supplementation [9, 16].

Prior to suspecting CD, the initial emergency management and subsequent dietary regimen for our patient followed standard UCD protocols, consisting of a high‐glucose infusion rate and low‐protein, carbohydrate‐rich diet. While this strategy effectively suppresses catabolism and reduces nitrogen load in classical UCDs, it proved deleterious due to the coexisting CD. In CD, this massive glucose load inevitably increases the cytosolic NADH/NAD+ ratio. This shift in redox state inhibits key glycolytic enzymes and impairs hepatic gluconeogenesis from lactate. Consequently, the patient suffered a dual metabolic insult: a massive lactate accumulation leading to profound lactic acidosis, alongside secondary mitochondrial energy failure that further exacerbated hyperammonemia [9]. Standard emergency measures for ASA thus triggered the fatal metabolic decompensation of CD.

Ultimately, this case highlights the critical need for individualized nutritional interventions guided by rapid genomic and biochemical profiling.

To navigate such therapeutic complexity in future similar cases, a tailored protocol should be implemented: (1) caloric support focused on medium‐chain triglycerides rather than high glucose loads; (2) cautious protein restriction supplemented with essential amino acids while avoiding high carbohydrate intakes; (3) meticulous monitoring of plasma ammonia, lactate, and quantitative amino acids; (4) early use of continuous hemodiafiltration/hemofiltration for ammonia clearance rather than aggressive dextrose‐driven hyperalimentation; and (5) prompt evaluation for liver transplantation, which represents the ultimate definitive cure for both metabolic disorders.

## 4 Conclusion

The present case highlights the diagnostic complexity of hyperammonemic disorders and underscores the importance of comprehensive molecular investigations in patients presenting with atypical phenotypes, particularly in populations with a high prevalence of consanguinity.

The coexistence of pathogenic variants affecting two genes involved in UC function is exceptionally rare and may result in a unique clinical and biochemical phenotype that cannot be fully explained by either disorder alone. This observation emphasizes the value of integrating detailed biochemical characterization with next‐generation sequencing technologies to unravel atypical UCDs, improve diagnostic accuracy, and facilitate the implementation of early, personalized nutritional and metabolic management strategies. Furthermore, it illustrates how multi‐locus pathogenic variation can modify disease expression and contribute to phenotypic complexity in inherited metabolic disorders.

## Author Contributions

M.Z. and M.B. collected the data and bibliography and drafted the manuscript. S.K., H.B.D., and A.B.C. contributed to patient care. J.H. performed metabolic and genetic investigations in Zurich. A.B.C. critically revised the collected data, reviewed the draft of the manuscript, and enhanced the discussion. M.S.A. supervised patient care and paper design. All authors read and approved the final manuscript.

## Funding

The authors have nothing to report.

## Ethics Statement

The study and the publication were approved by the Ethical local Committee of La Rabta Hospital.

## Consent

Informed consent for aminoacids profile analysis, genetic testing and publication was obtained from parents of the patient. Patient anonymity has been preserved.

## Conflicts of Interest

The authors declare no conflicts of interest.

## Supporting information


**Table S1:** Evolution of key biochemical parameters; NV: normal values.

## Data Availability

The data that support the findings of this study are available on request from the corresponding author. The data are not publicly available due to privacy or ethical restrictions.
