# Supplementary material for: Dual Urea Cycle Dysfunction Caused by Coexisting Argininosuccinic Aciduria and Citrin Deficiency: Clinical, Biochemical, and Molecular Insights From an Exceptional Case
Source: JIMD Rep. 2026 Oct 4;67(6):e70129. doi: 10.1002/jmd2.70129 (PMC13634694; doi:10.1002/jmd2.70129)
Supplement: Supplementary file 1 [file JMD2-67-e70129-s001.pdf]

	At presentation 30 months	Under high Carbohydrate, low protein diet	Acute decompensation 33 months
**Ammonia level (µmol/L) (NV<70 µmol/L)**	443	34.8→ 58.66	615
**Lactate (mmol/L) (NV :0.5-2.0 mmol/L)**	1.6	3.4 → 5.2	13.2
**pH/HCO3- (NV: 7.35-7.45 / 22-26 mmol/L)**	7.48 / 21.6	7,54 / 22,2	7.04/5.1

Supplementary Table 1: Evolution of key biochemical parameters; NV: normal values
